# Procrastination as a Marker of Anxiety Disorder Among College Students: An Institution-Based Cross-Sectional Study From Puducherry, India

**DOI:** 10.7759/cureus.61033

**Published:** 2024-05-25

**Authors:** Souriya Govindan, Mathan Kaliaperumal, M Arulmozhi, Padma Priya

**Affiliations:** 1 Psychiatry, Sri Venkateshwaraa Medical College Hospital & Research Centre, Pondicherry, IND; 2 Community Medicine, Sri Manakula Vinayagar Medical College and Hospital, Pondicherry, IND; 3 Clinical Psychology, SRM Institute of Science and Technology, Pondicherry, IND

**Keywords:** psychological marker, fear of failure, academic stress, hamilton anxiety scale, pass scale, anxiety, procrastination, mental health, college students

## Abstract

Background and objective

Procrastination, which refers to the act of unnecessarily delaying the beginning or completion of an assigned task, is a widespread but often neglected problem among college students. Postponing a task can impair academic performance or lead to stress and poor mental well-being. A knowledge gap exists in understanding the cyclical nature of the relationship between anxiety and procrastination, wherein anxiety leads to procrastination, which in turn exacerbates anxiety. In light of this, we aimed to assess the level of procrastination and anxiety among college students and to correlate the relationship between their procrastination and anxiety status.

Methodology

A cross-sectional analytical study was conducted among 347 graduate students from various disciplines at a tertiary care hospital in Puducherry, India. A convenient sampling technique was employed to recruit the participants, and data were collected using the validated Procrastination Assessment Scale for Students (PASS) and the Hamilton Anxiety Rating Scale (HAM-A).

Results

Our findings showed that about 124 (35.7%) participants reported weekly reading assignments as the most frequently procrastinated task, with a mean score of 3.15 ± 1.02, followed by studying for exams and writing term papers. The most commonly reported reason for procrastination was evaluation anxiety, followed by low self-esteem and perfectionism. A significant number of students (157, 45.2%) had mild anxiety, and 58 (16.7%) students were found to have severe anxiety. A Spearman correlation coefficient of 0.26 (95% CI: 0.16 - 0.36) was observed between the overall procrastination score and HAM-A score, which indicated a weak positive correlation and was statistically significant (p<0.05). Similarly, a weak positive correlation was found between task aversiveness, fear of failure, and anxiety levels.

Conclusions

Procrastination is positively related to anxiety but the relationship is weak. Understanding and addressing the underlying anxiety or stress can be a key to managing procrastination among college students.

## Introduction

Procrastination or task avoidance is a behavioral phenomenon, defined as a needless delay in beginning or completing tasks, which hinders an individual from reaching their goals [1]. In the context of education, academic procrastination refers to postponing assignments and coursework by the students, which unfortunately remains a neglected issue. It has been found that about 43% of college students procrastinate on their academic tasks in their day-to-day lives [2]. Procrastination exhibits a negative correlation with academic performance [3].

The most significant factors associated with procrastination behavior among college students are the desire for perfectionism, fear of failure, and task aversiveness [4]. Postponing a task often results in the need to rush to complete the task at the last minute and procrastinators frequently experience feelings of anxiety when faced with demanding responsibilities of task completion. Short-term academic procrastination is associated with late assignment submission, test anxiety, and underperformance, which in turn impact the mental health of the students [5,6]. Thus, procrastination results in anxiety, stress, and depression, leading to poor quality of life [7].

Anxiety is a multifaceted experience that encompasses worry, emotional instability, fear of failure, low self-esteem, and lack of confidence and peace of mind [8,9]. Anxiety among students may arise from factors such as last-minute studying, sleep deprivation, and poor time management. These stressors can lead to behaviors like mugging up for exams, fear of forgetting information, inadequate effort, guilt, and poor academic performance affecting class engagement [5]. Furthermore, students tend to procrastinate on work as a result of anxiety. While some element of worry is a necessary component of task-oriented behavior, excessive anxiety can be debilitating and adversely impact academic achievement [10]. Beyond learning, anxiety influences social interactions and physical and mental health [11]. Thus, these two components may have adverse effects on students' well-being and their academic performance. Understanding the consequences of procrastination is crucial, particularly in the field of academics. However, there is a lack of literature focusing on procrastination issues that could be a marker of anxiety disorder. Hence, we conducted this study to analyze the relationship between procrastination and anxiety among college students.

The primary objectives of our study are to assess the level of procrastination among college students by using the Procrastination Assessment Scale for Students (PASS) and to identify the reasons for procrastination in academic tasks. The secondary objectives are to determine the associated level of anxiety by using the Hamilton Anxiety Rating Scale (HAM-A) and to correlate the relationship between the student’s procrastination and their anxiety status.

## Materials and methods

Study design and participants

This cross-sectional analytical study was conducted in a tertiary care private medical college and hospital in Puducherry over six months from January 2022 to June 2022. Ethical approval was obtained from the Institutional Ethics Committee before the commencement of the study (approval no: 92/SVMCH/IEC-Cert/Nov 21). We included undergraduate or postgraduate students from medical and para-medical courses, aged 18-30 years, who were willing to participate in the study. Participants with preexisting psychological, neurological, learning, or other impairments that could interfere with their academic performance were excluded to ensure accurate measurement. Furthermore, participants diagnosed with any psychiatric disorders were offered confidential medical assistance, contingent upon their consent.

Based on the 43% prevalence of academic procrastination as reported in the study by Bhat et al. [2], with a 95% confidence interval (CI) and 6% absolute precision, the sample size was calculated to be 262 by using OpenEpi software. However, based on feasibility, we approached 373 participants, of which 26 declined to give consent, and recruited a total of 347 college students from various disciplines, by using a convenient sampling method.

Instruments and procedure

We collected data using a pre-designed, self-administered, semi-structured questionnaire from the participants, who fulfilled the inclusion criteria, without any known confounding comorbidities like substance use disorders, and psychiatric or medical comorbidities, which were ruled out clinically. To proceed with the study, the questionnaire underwent pilot testing to confirm its feasibility and understandability.

We conducted the study at the place and time of the participants' convenience. Before the participants completed the questionnaire, the researcher explained the research objectives to ensure autonomy and obtained informed consent. Participants were assured that their consent or withdrawal from the study would not affect their academic grades. Personal identifiers were avoided in the questionnaire to maintain confidentiality and eliminate information bias. Each participant took approximately 20-40 minutes to complete the questionnaire.

The sociodemographic details were collected using a standard semi-structured proforma. The level of procrastination was assessed using the PASS, developed by Solomon and Rothblum [12]. It has adequate construct-related validity and consists of 44 items in two parts. The first part comprises 18 items measuring procrastination levels in six academic areas: weekly reading assignments, studying for an exam, term paper writing, administrative tasks, attending meetings, and general academic tasks. There are three questions from each of these areas, rated on a 5-point Likert-type scale, assessing the frequency of procrastination on academic tasks, the degree to which task avoidance is problematic to students, and the student’s desire to reduce their procrastination. The second part consists of 26 items, each rated on a 5-point Likert-type scale ranging from 1 (not at all reflective of why I procrastinated) to 5 (definitely reflective of why I procrastinated). This section evaluates the reasons for academic procrastination with scores ranging from 26 to 130. The higher the score the greater the level of procrastination. The factor analysis examines the underlying reasons for procrastination in terms of fear of failure and task aversiveness.

The fear of failure scale is measured using five items with scores ranging from 5 to 25. The task aversiveness scale has three items, and scores range from 3 to 15. High scores suggest self-reported academic procrastination due to fear of failure and task aversiveness. The reliability estimates for the PASS measures are 0.84 for procrastination, 0.85 for fear of failure, and 0.76 for the task aversiveness factor [13].

The status of anxiety was assessed using HAM-A. It is a psychological rating scale, specifically designed to determine the intensity of anxiety symptoms in adults, adolescents, and children [14]. It comprises 14 items, each representing a group of symptoms. By utilizing the scale, we can measure both psychic-related anxiety, which includes mental agitation and psychological disturbances, as well as physical body-related anxiety. Each series of symptoms within the 14 items is rated on a scale of 0 to 4 in which a score of 4 indicates the highest severity. The scores from all these items are then combined to calculate a comprehensive score that reflects the individual’s overall severity of anxiety.

Statistical analysis

We employed SPSS statistics version 26.0 (IBM Corp., Armonk, NY) for data analysis, and a p-value less than 0.05 was considered statistically significant.

## Results

The study included a total of 347 participants. The mean age of the respondents was 23 ± 2 years. The largest proportion of participants (n=215) fell in the age group of 17-20 years, representing 61.96% of the total sample, whereas 107 participants (30.8%) were in the age group of 21-23 years, 17 (4.9%) under 24-26 years, and the remaining eight (2.3%) were more than 27 years of age. In terms of gender distribution, nearly three-fourths of the participants (256, 73.8%) were females, while 91 students (26.2%) identified as males; a substantial majority of the participants (327, 94.2%) participants were undergraduates and 20 (5.8%) were postgraduates. Based on the investigation into the history of psychiatric illness and substance abuse, about 13 participants (3.7%) reported a history of psychiatric illness, and six participants (1.7%) had a history of substance abuse. The results of the procrastination analysis are described under the following headings.

Frequency of procrastination

The PASS scale has categorized the frequency of procrastination across various academic tasks including writing term papers, studying for exams, and keeping up with weekly reading assignments, as well as academic administrative tasks, attendance, and school activities in general. Under each domain, the first two questions were used to compute the score, and a score of 4 or greater than 4 was used as a cut-off, indicating procrastination. In our study, about 124 (35.7%) participants reported weekly reading assignments as the most frequently procrastinated task with a mean score of 3.15 ± 1.02, followed by studying for the exams, which had a mean score of 3.03 ± 1.05 and was reported by 109 (31.4%) students. Writing a term paper was the third most procrastinated act (78, 22.5%) for students with a mean score of (2.90 ± 0.92). Academic administrative tasks like filling out forms, registration for classes, and getting ID cards had the lowest score of 2.2 ± 1.02, with only 43 (12.4%) students scoring 4 or higher (Table 1).

**Table 1:** Events frequently procrastinated by the participants (N=347) ^*^Multiple responses are possible as each respondent can choose more than one event of procrastination from a list SD: standard deviation

Sl. no	Areas of procrastination^*^	Procrastination score ≥4, n (%)	Mean procrastination score ± SD
1	Keeping up with weekly reading assignments	124 (35.7)	3.15 ± 1.02
2	Studying for exams	109 (31.4)	3.03 ± 1.05
3	Writing a term paper	78 (22.5)	2.90 ± 0.92
4	Attendance tasks (meeting the advisor, making appointments with the professor)	52 (14.9)	2.5 ± 1.45
5	School activities in general	49 (14.1)	2.3 ± 1.06
6	Academic administrative tasks (filling out forms, registering for classes, getting identity cards)	43 (12.4)	2.21 ± 1.02

Reasons for procrastination

Table 2 presents the most common reasons for procrastination by the participants concerning anxiety disorder. The reasons for procrastination were assessed using 26 questions, each scored on a range from 1 to 5, where 1 signifying “not at all reflects why I procrastinated” and 5 signifying “definitely reflects why I procrastinated”. The phrase “definitely reflects on procrastination” suggests that the respondent strongly agrees that the mentioned reason was a strong indicator of their procrastination behavior. These questions were grouped into 13 domains, each consisting of two questions. Then the average mean score of the two questions for each domain was calculated. The major domains included perfectionism, evaluation anxiety, low self-esteem, task aversiveness, laziness, time management, difficulty making decisions, peer pressure, dependency, lack of assertion, risk-taking, fear of success, and rebellion against control. Among these domains, evaluation anxiety emerged as the most common reason for procrastination, followed by perfectionism and difficulty in making decisions. Of the students who reported evaluation anxiety, a significant proportion (99, 28.5%) identified it as a definite reason for their procrastination.

**Table 2:** Reasons for procrastination reported by the students (N=347) SD: standard deviation

Sl. no	Major domains of procrastination	Sub-domains	Score, mean ± SD
1	Evaluation anxiety	Worried about getting a bad grade	3.20 ± 1.51
		Concerned professor wouldn’t like your work	2.92 ± 1.41
2	Perfectionism	Set very high standards yourself and worry that won’t be able to meet those standards	3.03 ± 1.51
		Concerned you won’t meet your own expectations	3.03 ± 1.45
3	Difficulty making decisions	Hard time knowing what to include and not to include in your paper	3.19 ± 1.34
		Couldn’t choose among all the topics	2.80 ± 1.29
4	Laziness	Didn’t have enough energy to begin the task	2.88 ± 1.49
		Just felt too lazy to write a term paper	3.02 ± 1.48
5	Time management	Had too many other things to do	3.14 ± 1.44
		Felt overwhelmed by the task	2.73 ± 1.28
6	Lack of assertion	Need to ask the professors for information, but felt uncomfortable approaching them	3.05 ± 1.41
		Difficulty in requesting information from other people	2.78 ± 1.44
7	Dependency	Waited to see if the professor would give some more information about the paper	2.71 ± 1.41
		Waited until a classmate did his/hers to get some advice	2.94 ± 1.39
8	Task aversion	Disliked writing a term paper	2.70 ± 1.47
		Felt too long to write a term paper	2.81 ± 1.34
9	Low self-esteem	Didn’t trust yourself to do a good job	2.46 ±1.49
		Didn’t think you knew enough to write the paper	2.85 ± 1.37
10	Risk-taking	Looked for the excitement of risk-taking this task at the last minute	2.68 ± 1.51
		Liked the challenge of waiting until the deadline	2.55 ± 1.56
11	Rebellion against control	Resented people setting deadlines for you	2.52 ± 1.42
		Resented to do things assigned by others	2.63 ± 1.38
12	Peer pressure	Knew that your classmates hadn’t started the paper either	2.78 ± 1.42
		Friends were pressuring you to do other things	2.37 ± 1.41
13	Fear of failure	Concerned that if you got a good grade, people would have higher expectations of you in the future	2.60 ± 1.49
		Concerned that if you did well, your classmates would resent you	2.31± 1.39

Grading of anxiety

Based on the scores of HAM-A, out of 347 participants, about 157 (45.2%) had mild anxiety, 64 (18.4%) were categorized as having mild to moderate anxiety, 55 (15.9%) had moderate anxiety, whereas 58 (16.7%) students had severe anxiety (Table 3).

**Table 3:** Grade of anxiety among study participants according to Hamilton Anxiety Rating Scale (N=347)

Grade of anxiety with scores	N (%)
Not present (0)	13 (3.8)
Mild severity (1-17)	157 (45.2)
Mild to moderate severity (18-24)	64 (18.4)
Moderate severity (25-30)	55 (15.9)
Severe (more than 30)	58 (16.7)

The fear of failure was calculated by taking the mean scores of the sum of questions 19, 24, 33, 39, and 42, and task aversiveness was determined by computing the mean sum of questions 27, 34, and 35. The mean total score for fear of failure was 1016.5 (2.9 ± 0.9) while it was 970.3 (2.8 ± 1.01) for task aversiveness. We calculated its correlation with anxiety by computing the total HAM-A scores and the above scores for fear of failure and task aversiveness. The findings revealed a weak positive correlation of 0.28 between fear of failure and anxiety. Similarly, a weak positive correlation of 0.27 was found between task aversiveness and anxiety. (p<0.05).

**Figure 1:**
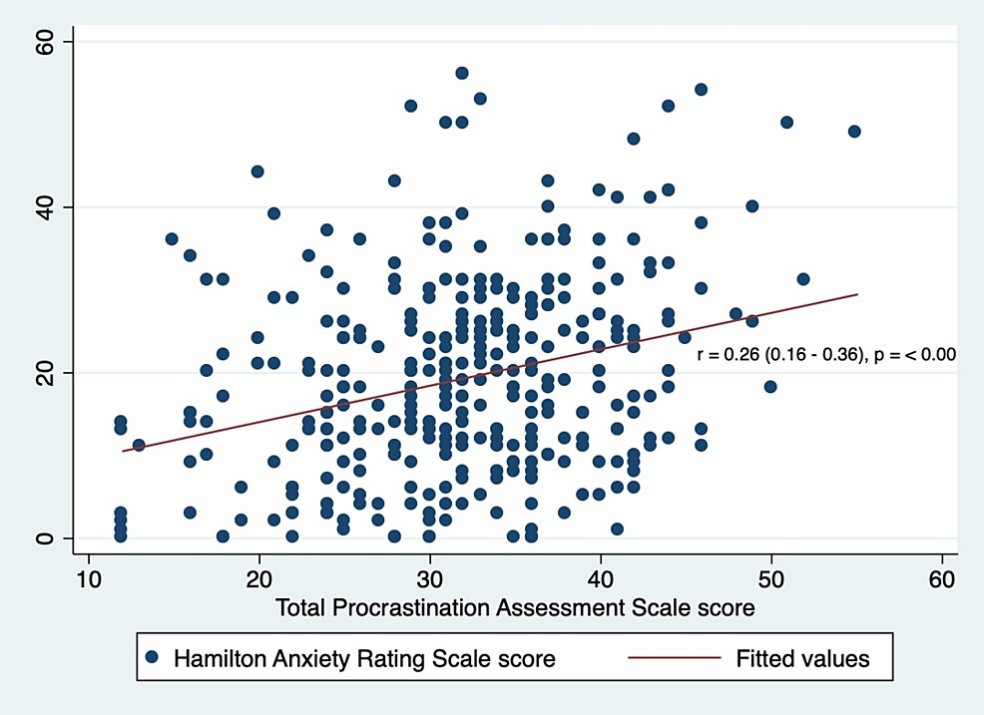
Correlation between total procrastination score and Hamilton Anxiety Rating Scale score r: Spearman correlation coefficient

In the final analysis, examining the relationship between student procrastination and their anxiety levels, a Spearman correlation coefficient of 0.26 (95% CI: 0.16 - 0.36) was observed between the overall procrastination score and the HAM-A score. This indicates a weak positive correlation, which was statistically significant with a p-value less than 0.05 (Figure 1).

## Discussion

Our findings showed that among all academic tasks, weekly reading assignments had higher procrastination scores compared to other parameters, which aligns with the results of Custer et al. [15]. However, in the studies conducted by Solomon et al. [12] and Onwuegiebuzi et al. [16], writing term papers was the most frequently procrastinated act followed by weekly reading assignments. This might be due to participants not perceiving weekly reading assignments as significant or as directly contributing to their grades compared to summative tests. Furthermore, evaluation anxiety was the most common reason for procrastination, which is consistent with previous study findings [17]. Hence, discussing the purpose of evaluation and showing the academic results in private, accompanied by effective feedback for improvement can reduce evaluation anxiety [18].

Even though our study showed a weak positive correlation between procrastination and anxiety, our findings align with the study conducted by Desai et al. [5]. The relationship between procrastination and anxiety is complex and it is hard to understand whether procrastination is a result or driving force of anxiety. While some studies have demonstrated a correlation between anxiety and procrastination, others have not identified anxiety as a cause of procrastination. A study by Cassady and Johnson [19] found that students with high levels of anxiety tend to procrastinate more than their counterparts. Another study conducted by Saplaskwa et al. [20] showed that students with high procrastination levels were more prone to experience anxiety. Of note, Uzun Ozer et al. [21] found no significant relationship between procrastination and anxiety levels. In general, as per evidence, it can be stated that procrastination serves as a self-protective strategy for different types of anxiety, which in turn significantly influences the frequency of procrastination. Therefore, procrastination can stem from different forms of anxiety or by delaying assigned tasks.

The correlation of both variables might be poor due to the young age of the population studied from a medical background, and anxiety disorders often develop over the course of life. Additionally, individual responses to anxiety can vary significantly Other factors such as personality traits, study habits, time management skills, and academic self-efficacy could also influence procrastination behavior [22]. The results might differ across various study populations, including children and working adults, and in different cultural contexts due to varying stressors and coping mechanisms. We found a weak positive correlation between fear of failure and anxiety, leading to academic procrastination. Similarly, a study by Sudirman et al. [4] found a positive association between fear of failure and academic procrastination. Conversely, the fear of not meeting the expected standards can deter individuals from initiating or completing tasks, whereas a small amount of fear can also serve as a motivator to accomplish academic tasks.

Our study also showed a weak correlation between task aversiveness and anxiety, which ultimately leads to academic procrastination. In a study conducted by Steel et al. [23], the potential benefits of procrastination have been explored by differentiating between passive and active procrastinators. It was found that passive procrastinators delay tasks due to negative emotions such as anxiety and indecisiveness, which validates our study findings. On the other hand, active procrastinators intentionally defer tasks, finding it more comfortable to complete them under the pressure of tight deadlines.

Our study uniquely integrates the reasons for procrastination with the correlation between anxiety and procrastination among college students. However, it has a few limitations. We employed a cross-sectional design, which restricted us from establishing cause-effect relationships. Moreover, the study was conducted at a single center by using non-probability sampling, which may limit the generalizability of the results. Furthermore, the weak correlation between procrastination and anxiety levels was assessed only with the HAM-A scale, which might not fully evaluate the hypothesis. To strengthen the evidence on the association between anxiety disorder and procrastination, a follow-up study with a larger sample size in the community setting is recommended.

## Conclusions

Overall, about one-third of college students procrastinate various academic tasks and nearly half of the population experiences anxiety. Keeping up with weekly assignments was the task most commonly associated with procrastination, often due to evaluation anxiety. A linear relationship exists between anxiety level and academic procrastination, i.e., as anxiety increases, the tendency to delay tasks also increases, even though the strength of the relationship remains poor. Factors such as anxiety, fear of failure, and task aversiveness are all interconnected, and all these can influence a student’s tendency to delay tasks. Hence, recognizing the psychological factors is critical in addressing procrastination among students. Despite the weak correlations, our findings shed light on the potential mental health implications of procrastination. Therefore, promoting effective learning strategies, stress management techniques, and time management skills through a robust student support system can significantly enhance students' learning outcomes and their overall well-being.
